# Use of *Ligilactobacillus salivarius* SP36 as an Adjunct Culture by an Artisan Dairy and Isolation of New Autochthonous Strains with Technological Potential for Cheesemaking

**DOI:** 10.3390/foods15081362

**Published:** 2026-04-14

**Authors:** Josué Jara, Claudio Alba, Javier Calzada, Lucía Largo, Marta Kellermann, Sara Rosado, Marta Ávila, Sonia Garde, Juan M. Rodríguez

**Affiliations:** 1 Department of Galenic Pharmacy and Food Technology, Complutense University of Madrid, 28040 Madrid, Spain; lulargo@ucm.es (L.L.); martakel@ucm.es (M.K.); 2 Instituto Pluridisciplinar, Complutense University of Madrid, 28040 Madrid, Spain; c.alba@ucm.es; 3 Department of Nutrition and Food Science, Complutense University of Madrid, 28040 Madrid, Spain; 4 Departamento de Tecnología de Alimentos, Instituto Nacional de Investigación y Tecnología Agraria y Alimentaria (INIA-CSIC), Carretera de La Coruña km 7, 28040 Madrid, Spain; jcalzada@inia.csic.es (J.C.); arribas@inia.csic.es (M.Á.); sgarde@inia.csic.es (S.G.); 5 Quesería Siricueta, 22347 Morillo de Sampietro, Spain; sararosado@hotmail.com

**Keywords:** cheese, *Ligilactobacillus salivarius*, *Lactiplantibacillus plantarum*, *Lacticaseibacillus paracasei*, adjunct cultures, cheese technology, cheese safety

## Abstract

Artisanal cheese quality relies on a complex microbiota. The generalized use of commercial starter cultures has been associated with reduced microbial diversity, fueling interest in using indigenous lactic acid bacteria (LAB) as adjunct cultures. This study aimed to evaluate *Ligilactobacillus salivarius* SP36 as a starter or adjunct culture in ripened cheeses. Culture-based and culture-independent analyses were performed, together with the assessment of some physico-chemical parameters (pH, water activity, and color), including the profile of volatile compounds. All cheeses were microbiologically safe according to current EU legislation. The pH of the cheese made only with the SP36 strain was higher than those of the cheeses manufactured with a commercial starter (with or without strain SP36). *L. salivarius* SP36 modulated the aroma profile by increasing ethyl esters, alcohols, ketones, organic acids and sulphur compounds. LAB dominated all cheeses, with the highest microbial diversity in the cheese produced without the commercial starter. *Lactiplantibacillus plantarum* and *Lacticaseibacillus paracasei* isolates were obtained from all cheeses. Overall, *L. salivarius* SP36 seems a promising adjunct for mature cheeses, while autochthonous *L. plantarum* and *L. paracasei* isolates represent promising candidates for starter or adjunct cultures.

## 1. Introduction

Artisanal cheeses produced from raw milk are highly valued worldwide due to their nutritional contribution, distinctive sensory characteristics, and close association with cultural and gastronomic heritage [1]. Their complexity arises from a diverse microbiota that originates from raw milk, the cheesemaking environment, and natural fermentations, which together play a critical role in shaping cheese physico-chemical properties, flavor, and safety [2,3]. Traditionally, studies on cheese microbiota have relied on culture-dependent techniques, which, although informative, provide a limited perspective of the microbial consortia inhabiting these ecosystems. Recent advances in high-throughput sequencing have allowed deeper insights into the microbial community structure and succession during cheese ripening [4,5,6,7]. These tools are transforming the study of artisanal cheeses, offering a more comprehensive understanding of their microbial ecosystems.

Although the use of selected starter cultures has improved standardization and safety in cheesemaking, this practice can reduce microbial diversity and attenuate the distinctive character of artisanal cheeses [8,9]. Flavor development in cheese is closely linked to microbial metabolism [10]. Lactic acid bacteria (LAB) and secondary microbiota drive proteolysis, lipolysis, and amino acid catabolism, generating a broad spectrum of volatile compounds that contribute to sensory identity [11,12,13,14]. Preserving this microbial biodiversity is not only essential for maintaining product typicity but also contributes to ecological balance, as beneficial microbial consortia can exert antagonistic effects against spoilage and pathogenic microorganisms [15,16]. For this reason, there is increasing interest in exploring autochthonous LAB as adjunct cultures that can contribute both technological and probiotic functionalities [16,17]. Among them, some *Ligilactobacillus salivarius* strains have drawn attention due to their biopreservative, technological and sensorial properties, positioning them as promising candidates for dairy applications [18,19]. Recent studies have demonstrated that such *L. salivarius* strains can influence cheese ripening, modulate volatile profiles, and enhance flavor development, highlighting their potential as adjunct cultures in artisanal facilities [20].

In a previous work, we isolated the strain *L. salivarius* SP36 from an old cheese seal that was used from 1877 to 1936 to label the cheeses elaborated in a small dairy in the Spanish Pyrenees [21]. Subsequently, we demonstrated that the strain had the potential to be employed as an adjunct culture in cheesemaking, affecting positively the organoleptic properties of the cheeses [22]. The aim of this study was to assess the effect of *L. salivarius* SP36, either alone or in combination with a commercial starter culture, in a small artisanal dairy whose location shares the same ecological conditions as the dairy from which this strain was originally isolated. For this purpose, the physico-chemical (including volatile compound profile) and microbiological characteristics of the cheeses were assessed.

## 2. Materials and Methods

### 2.1. Cheese Manufacture

Three types of experimental raw milk cheeses (~1 kg each) were produced in the artisanal dairy Quesería Siricueta (Morillo de Sampietro, Huesca, Spain). The milk was obtained from sheep and goats (9:1 ratio), manually milked and filtered. The batches (40 L) were elaborated according to the European Guide to Good Hygiene Practices for the Production of Artisanal Cheeses and Dairy Products [23].

The three types of cheese were: (i) C1 (control): milk inoculated (1 g/40 L) with the commercial starter Aromático tipo B (Abiasa, Tui, Spain), containing *Lactococcus lactis* subsp. *lactis*, *L. lactis* subsp. *cremoris* and *L. lactis* subsp. *lactis* biovar. diacetylactis; (ii) C2: same commercial starter plus *L. salivarius* SP36 (50 g containing ~9 log_10_ CFU/g in 40 L); and (iii) C3: only *L. salivarius* SP36 (same inoculum dose). Three units of each type of cheese were produced. Ovine rennet (75 rennet units; Cuajos Caporal, Cistérniga, Spain) was added according to the manufacturer’s instructions. After coagulation, the curd was cut, heated to 38 °C while stirred, drained, and placed in molds. The cheeses were ripened at the dairy facilities for 150 days under controlled temperature and humidity conditions. After the ripening period, three different samples (~100 g each) of each cheese were collected for subsequent analyses.

### 2.2. Physico-Chemical Analyses of Cheeses

The physico-chemical characterization of cheeses included the determination of pH, water activity (a_w_), and color. Three samples of each cheese were used in the pH and a_w_ assays, while seven samples were employed for determining color coordinates. For the pH analysis, 25 g of cheese was homogenized in 225 mL of sterile peptone water (1:10, *w*/*w*) using a Stomacher (IUL Instruments, Barcelona, Spain), and measurements were taken directly in the homogenate with a pH meter (HI98161, Hanna Instruments, Eibar, Spain). Water activity (a_w_) was assessed with an Aqualab 4TE device (Meter Group, Pullman, WA, USA) after temperature equilibration. Color was measured directly on the cheese surface using a Chromameter CR400 (Konica Minolta, Tokyo, Japan), and the CIELAB coordinates L*, a*, and b* were recorded. Each parameter was determined in triplicate, except for color, which was measured in seven replicates per sample.

### 2.3. Analysis of Volatile Compounds

The cheese pieces (two portions of two cheeses for each cheese type) were wrapped in aluminum foil, vacuum-packed, and stored at −40 °C after 150 d of ripening, until analysis. Volatile compounds of cheeses were extracted by automated solid-phase microextraction (SPME) and analyzed by gas chromatography–mass spectrometry (GC-MS) as described by Arias et al. [22]. All determinations were performed in duplicate. Relative abundances of volatile compounds were expressed as the percentage of each peak area relative to that of the internal standard (cyclohexanone).

The SPME conditions were as follows: The cheese portions (10 g) were homogenized in a mechanical grinder with 15 g of Na_2_SO_4_ and 30 μL of an aqueous solution of cyclohexanone. Five grams of the mixture was weighed in a 20 mL headspace glass vial sealed with a PTFE-faced silicone septum (Supelco, Bellefonte, PA, USA). The vials were placed on an autosampler tray and subjected to SPME. Both the equilibration and extraction phases were carried out at 37 °C for 20 and 30 min, respectively. A 2 cm 50/30 mm StableFlex Divinylbenzene/Carboxen/Polydimethylsiloxane (DVB/CAR/PDMS)-coated fiber (Supelco) was used for headspace extraction. Desorption into the GC injection port was done at 260 °C for 10 min in splitless mode. Before use, the fiber was conditioned in the GC injection port at 270 °C for 1 h as recommended by the manufacturer.

### 2.4. Culture-Based Microbiological Analysis

The microbiological safety of the cheeses was evaluated by a culture-based analysis, aimed toward the isolation and enumeration of the microorganisms required by the current European Union (EU) legislation [Regulation [EC] No. 2073/2005]: *Salmonella* spp., *Listeria monocytogenes* and coagulase-positive staphylococci. Samples (25 g) from all the cheeses were homogenized in 225 mL sterile peptone water (1:10, *w*/*w*); serial dilutions were prepared in peptone water, and inoculated on different selective media that allow the growth of the cited bacteria: Salmonella–Shigella agar (SS, Scharlab, Barcelona, Spain) for *Salmonella* spp., Brilliance Staph agar (Biomerieux, Marcy-L’Etoile, France) for staphylococci, and Brilliance Listeria agar (Oxoid, Basingstoke, UK) for *Listeria monocytogenes*. In addition, MacConkey agar (Oxoid) plates were used for the potential isolation of other enterobacteria, including coliforms. Finally, plates of MRS agar (Oxoid) supplemented with cysteine (0.25% *w*/*v*) and bromophenol blue (16 mg/L) were also included for the isolation of autochthonous non-starter LAB.

The plates were incubated at 37 °C for 48 h in aerobiosis. Each distinct colony morphology was counted, and a representative colony of each type was streaked onto BHI agar (Oxoid) to obtain pure cultures after a 37 °C overnight incubation. From these pure streaks, a single colony was transferred into BHI broth and incubated at 37 °C for 24 h. These cultures were then preserved as frozen stocks in BHI broth (Oxoid) supplemented with 30% glycerol at −20 °C for subsequent analyses.

### 2.5. Identification of the Isolates

Genomic DNA was extracted from pure isolates following Baele et al. [24]. PCR amplification of the 16S rRNA gene was performed using primers ANLACTO1 (AGAGTTTGATCCTGGCTCAG) and ANLACTO2 (GGCTGCTGGCACGTAGTTAG). Reactions were carried out in a thermocycler (Bio-Rad, Hercules, CA, USA) under standard conditions. Amplicons were visualized by electrophoresis (1.2% agarose gel, 90 V, 40 min), stained with GelRed (Biotium, Fremont, CA, USA). PCR products were purified using a commercial kit (NucleoSpin^®^ Gel and PCR Clean-up, Macherey-Nagel, Allentown, PA, USA) and sequenced at STABVIDA (Caparica, Portugal). Taxonomic affiliation was assigned by BLASTN (NCBI Core nt database). Later, identification of the LAB isolates was confirmed by MALDI-TOF mass spectrometry as described previously [25].

### 2.6. Quantification of L. salivarius DNA in the Cheese Samples

The concentration of *L. salivarius* DNA in the cheese samples was estimated using a real-time quantitative PCR assay following the procedure of Harrow et al. [26] and adapted by Fernández et al. [27], which is based on the amplification of a 97 bp product of the 16S–23S intergenic spacer region of *L. salivarius*. Samples of DNA extracted from milk cultures containing known concentrations of *L. salivarius* SP36 (from 1 to 9 log_10_ CFU/g) were used as the standard curve.

### 2.7. Antibiotic Susceptibility Testing

The antibiotic susceptibility of the LAB isolates obtained from the cheeses was determined by E-test (Biomerieux, France). Strains were inoculated into BHI agar (10^4^ CFU/mL) and incubated at 37 °C for 48 h. Minimum inhibitory concentrations (MICs) were determined for gentamicin, tetracycline, erythromycin, kanamycin, clindamycin, streptomycin, vancomycin, chloramphenicol, and ampicillin, following EFSA guidelines [28].

### 2.8. Biosynthesis of Biogenic Amines

The ability of the LAB *Enterococcus lactis* and *Escherichia fergusonii* isolates to form biogenic amines (tyramine, histamine, putrescine and cadaverine) by decarboxylation of the precursor amino acids (tyrosine, histidine, ornithine and lysine, respectively) was qualitatively assessed using the method described by Bover-Cid and Holzapfel [29]. The precursor amino acids were purchased from Sigma-Aldrich (St. Louis, MO, USA).

### 2.9. Whole Genome Sequencing (WGS) of L. plantarum Q132 and L. paracasei Q133 and Preliminary Genome Mining

Cultures of the *L. plantarum* Q132 and *L. paracasei* Q133 strains were grown in MRS broth for approx. 16 h at 37 °C. Bacterial cells were harvested by centrifugation (1.5 mL at 13,000 rpm for 2 min). The pellets were resuspended in 1 mL of TE buffer (10 mM Tris-HCl, pH 8.0, 1 mM EDTA), centrifuged again, and the final pellets were frozen at −20 °C. DNA was extracted using the Quick-DNA™ Fungal/Bacterial Miniprep Kit (Zymo Research, Irvine, CA, USA) according to the manufacturer’s instructions.

WGS libraries were prepared using a hybrid sequencing approach, comprising independent library preparation protocols for Oxford Nanopore long-read sequencing and Illumina short-read sequencing. For Oxford Nanopore sequencing, libraries were prepared using an amplification-free long-read library preparation protocol based on Oxford Nanopore Technologies v14 chemistry, following the manufacturer’s instructions as implemented by the sequencing service provider. Library preparation included sequence-independent DNA fragmentation via transposase-mediated tagmentation, applying minimal fragmentation of the input genomic DNA. No PCR amplification step was performed during Oxford Nanopore library construction.

For the Oxford Nanopore library, DNA fragmentation was not performed by size-based selection. Fragmentation was achieved via sequence-independent tagmentation, and no size-selection step was applied during library preparation. As a result, small plasmids and other low-molecular-weight extrachromosomal elements were not selectively excluded during Oxford Nanopore library construction. For the Illumina library, DNA fragmentation was performed according to standard Illumina library preparation procedures. The Illumina sequencing data were used exclusively for polishing the long-read assembly and not for independent genome reconstruction.

Oxford Nanopore sequencing was performed using R10.4.1 flow cells and primer-free sequencing protocols. Basecalling of the Oxford Nanopore raw signal data was performed using Dorado Super-Accurate basecalling, applying default quality filtering parameters as implemented by the sequencing service provider. Illumina sequencing was performed using standard Illumina instrumentation, generating paired-end reads of 2 × 150 bp.

Quality filtering and trimming were performed as part of the sequencing data processing pipeline. Oxford Nanopore reads were basecalled using Dorado Super-Accurate mode with default Q10 quality filtering, followed by the removal of the lowest-quality 5% of reads using Filtlong v0.2.1 (default parameters). Illumina reads were quality filtered and trimmed using fastp with default parameters, including adapter trimming, the removal of low-quality bases (Phred score threshold ≥ 15), and the removal of reads shorter than 50 bp.

A genome-based safety assessment was performed using the assembled draft genomes of both strains. Structural and functional annotation were independently conducted using Bakta (v1.9.3) [30] with its corresponding database release, and the RAST server (RASTtk; accessed [March 2026]) [31], both run under default parameters. The resulting annotation outputs were subsequently integrated to generate a consolidated annotated dataset for each genome. The whole genome sequences of *L. plantarum* Q132 and *L. paracasei* Q133 were deposited in GenBank under accession number PRJNA1442380.

The presence of genes encoding classical foodborne toxins was evaluated through a systematic interrogation of the combined Bakta and RAST annotations, focusing on curated virulence-associated features. The potential for biogenic amine production was assessed by screening for the canonical gene clusters involved in histamine and tyramine biosynthesis, specifically targeting histidine decarboxylase (*hdc*) and tyrosine decarboxylase (*tdc*) systems, based on the combined annotation dataset. Antimicrobial resistance determinants were identified using the Resistance Gene Identifier (RGI v6.0.5) [32] against the Comprehensive Antibiotic Resistance Database (CARD v4.0.1) [33]. Only hits classified as “strict”, based on CARD-curated bit-score cut-offs, were retained for downstream analysis, while “loose” hits were excluded to ensure high-confidence detection.

### 2.10. Metataxonomic Analysis of the Cheeses

A metataxonomic analysis was performed on the cheese samples (three samples from each of the three types of cheeses) collected at day 150 of ripening. DNA was extracted from the homogenized cheese samples using the commercial kit QIAamp^®^ DNA Mini Kit 250 (Qiagen, Hilden, Germany), following the manufacturer’s protocol. The amplification of the bacterial 16S rRNA gene targeted the V3–V4 hypervariable region using a dual-barcoded two-step PCR. Equimolar concentrations of the universal primers S-D-Bact-0341-b-S-17 (5′-ACACTGACGACATGGTTCTACACCTACGGGNGGCWGCAG-3′) and S-D-Bact-0785-a-A-21 (5′-TACGGTAGCAGAGACTTGGTCTGACTACHVGGGTATCTAATCC-3′), with Illumina adapter overhangs, were used. Barcodes were appended to the PCR amplicons to allow the separation of forward and reverse sequences.

The concentration of PCR products was determined using a 2100 Bioanalyzer (Agilent, Santa Clara, CA, USA). Barcoded products were pooled at approximately equimolar concentrations and run on a preparative agarose gel, and the correct band size was excised and purified using a QIAEX II Gel Extraction Kit (Qiagen). The pooled amplicons were quantified with PicoGreen (BMG Labtech, Jena, Germany) and sequenced on an Illumina MiSeq platform (2 × 300 bp paired-end reads) at the facilities of the Scientific Park of Madrid (Spain).

The raw sequences were processed in QIIME2. Quality filtering, denoising, and chimera removal were performed with DADA2, and amplicon sequence variants (ASVs) were taxonomically assigned using the naïve Bayes classifier trained against the SILVA 138.1 reference database. A feature table containing ASVs per sample was generated and normalized by total sum scaling (TSS), dividing each ASV count by the total library size to obtain relative abundances.

### 2.11. Statistical Analysis

Microbial data were expressed as log_10_ CFU/mL. Statistical analyses and plotting were performed using the R software (×64) version 4.0.3 (desktop). Variables were reported as means ± standard deviation (SD). ANOVAs were performed for parametric data. The statistical significance was set at *p*-value < 0.05, with the exception of the analysis of volatile compounds where it was set at *p* < 0.01 because of the critical relevance of such compounds for some sensorial properties of ripened cheeses. A principal component analysis (PCA) with Varimax rotation was carried out on volatile compounds that showed significant differences among cheeses.

## 3. Results

### 3.1. Physico-Chemical Characteristics of Cheeses

The physico-chemical parameters of the cheeses after 150 days of ripening are shown in Table 1. The pH values differed when the C3 cheeses were compared to the C1 and C2 cheeses, with C3 (6.35 ± 0.10) showing higher values than C1 (5.69 ± 0.05) and C2 (5.58 ± 0.09) (*p* = 0.0001). Water activity (a_w_) values were close to 0.9 in all cheeses but C3 presented the lowest value (0.88 ± 0.003), which was significantly different from C1 (0.91 ± 0.006) and C2 (0.90 ± 0.004) (*p* = 0.0006). Regarding color, lightness (L*) was significantly lower in C2 (67.89 ± 4.18) compared to C1 (75.65 ± 1.56) and C3 (75.09 ± 2.90) (*p* = 0.0002). The a* coordinate (red/green) also differed significantly, with C2 (−5.09 ± 0.48) showing lower values than C1 (−4.38 ± 0.25) and C3 (−4.39 ± 0.19) (*p* = 0.005). In contrast, the b* coordinate (yellow/blue) did not differ significantly among cheeses (*p* = 0.1489), with values ranging from 21.09 ± 1.20 in C2 to 21.87 ± 0.63 in C3.

### 3.2. Analysis of Volatile Compounds

The volatile fraction of the cheeses was characterized by 51 volatile compounds identified by SPME/GC-MS, including eight hydrocarbons, two aldehydes, six ketones, eight esters, 13 alcohols, three sulphur compounds, one terpene and 10 carboxylic acids. The addition of *L. salivarius* SP36 to cheese milk significantly (*p* < 0.01) increased the formation of 28 volatile compounds and decreased that of one compound (Table 2). More specifically, the presence of *L. salivarius* favored the formation of six ethyl esters, whose levels were higher in the cheese made with both the commercial starter and the *L. salivarius* strain (C2), except for ethyl-2-methylbutyrate, which showed a higher relative abundance in the cheese manufactured with *L. salivarius* alone (C3) (Table 2).

In addition, the C2 cheese showed higher (*p* < 0.01) concentrations of 2-propanol, ethanol, 2-pentanol, 1-butanol, 2-heptanol and 1,3-butanediol than the other two cheeses (Table 2). The levels of 2-methyl-1-propanol, 1-methoxy-2-propanol and 3-methyl-1-butanol were significantly higher in the cheese made only with the *L. salivarius* strain (C3) than in the control cheese (C1). On the other hand, the relative abundance of 2,3-butanediol was higher in the two cheeses that were manufactured including *L. salivarius* SP36 (C2 and C3) (Table 2). Conversely, the C1 cheese (with only the commercial starter) exhibited higher levels of phenyl ethanol than the other two cheeses incorporating *L. salivarius* (C2 and C3).

**Figure 1:**
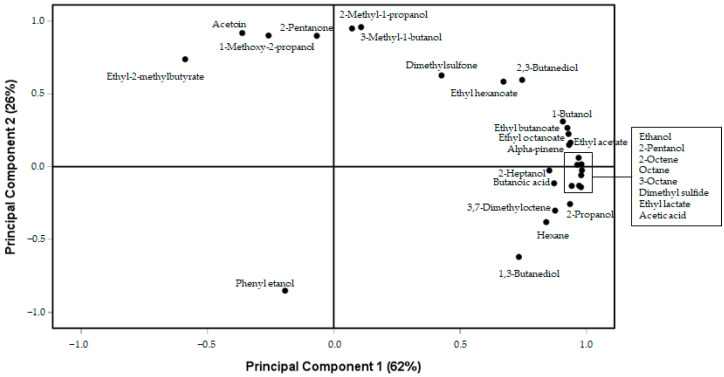
PCA showing the first two principal components of selected volatile compounds.

**Figure 2:**
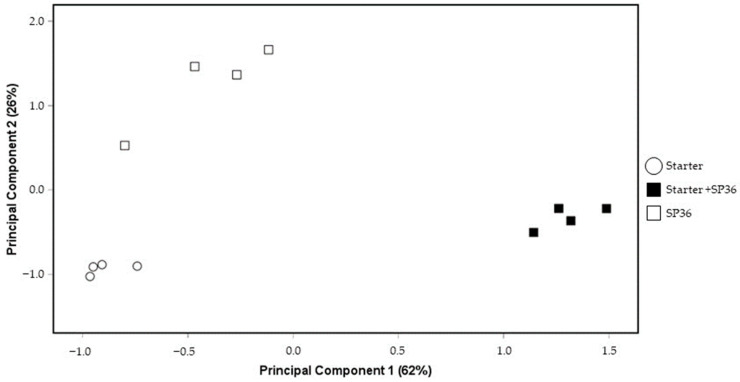
Distribution of cheeses using principal components 1 and 2.

The C3 cheese showed significantly (*p* < 0.01) higher levels of 2-pentanone and acetoin (Table 2). The use of *L. salivarius* as an adjunct in C2 cheese manufacture also promoted (*p* < 0.01) the formation of acetic and butanoic acids. Levels of dimethyl sulfide were higher (*p* < 0.01) in the C2 cheese, while dimethyl sulfone levels were higher in the two cheeses including *L. salivarius* SP36 (C2 and C3) (Table 2). Finally, the C2 cheese showed higher (*p* < 0.01) levels of α-pinene than the other two cheeses. The principal component analysis (PCA) is shown in Figure 1 and Figure 2. Figure 1 represents the loading plots of the variables in the plane defined by principal components 1 (PC1) and 2 (PC2), which explained 62 and 26% of the variance, respectively. Ethanol, 2-pentanol, 2-octene, octane, 3-octane, dimethyl sulfide, ethyl lactate, acetic acid, ethyl acetate, 2-propanol, alpha-pinene, ethyl octanoate, ethyl butanoate, 1-butanol, 3,7-dimethyloctene, butanoic acid, 2-heptanol, hexane, 2,3-butanediol, 1,3-butanediol and ethyl hexanoate correlated positively with PC1, whereas ethyl-2-methylbutyrate correlated negatively. On the other hand, 3-methyl-1-butanol, 2-methyl-1-propanol, acetoin, 2-pentanone, 1-methoxy-2-propanol, ethyl-2-methylbutyrate, dimethylsulfone, 2,3-butanediol and ethyl hexanoate correlated positively with PC2, whereas phenyl ethanol and 1,3-butanediol correlated negatively.

Figure 2 shows the loading plots of the factor scores for each cheese sample. PC1 separated the cheeses made with the starter and SP36 from the rest of the cheeses. However, PC2 separated the cheeses only made with SP36 from the other two cheeses.

### 3.3. Isolation, Identification and Characterization of Bacteria

All cheeses complied with the microbiological criteria established by Regulation (EC) N° 2073/2005 of the European Union for this type of food since *Listeria monocytogenes*, coagulase-positive *Staphylococcus*, and *Salmonella* spp. were not detected in any of the samples, confirming the hygienic adequacy of the manufacturing process. A low bacterial concentration ranging from 2.73 to 3.41 log_10_ CFU/g was observed on the Brilliance Listeria agar plates seeded with samples from the C1 and C3 cheeses, but none of them were identified as *L. monocytogenes*. Similarly, a low bacterial population, ranging from 1.60 to 3.41 log_10_ CFU/g, was observed on the Brilliance Staph 24 agar from the three types of cheeses, but none of them were identified as coagulase-positive *Staphylococcus*. No bacterial growth was detected on the Salmonella–Shigella plates. Growth on the MacConkey plates (~3.10 log_10_ CFU/g) was only detected from the C3 samples, although none of the colonies belonged to the genus *Salmonella*. In contrast, high LAB counts were recorded in all cheeses, ranging from 4.87 to 6.77 log_10_ CFU/g.

**Figure 3:**
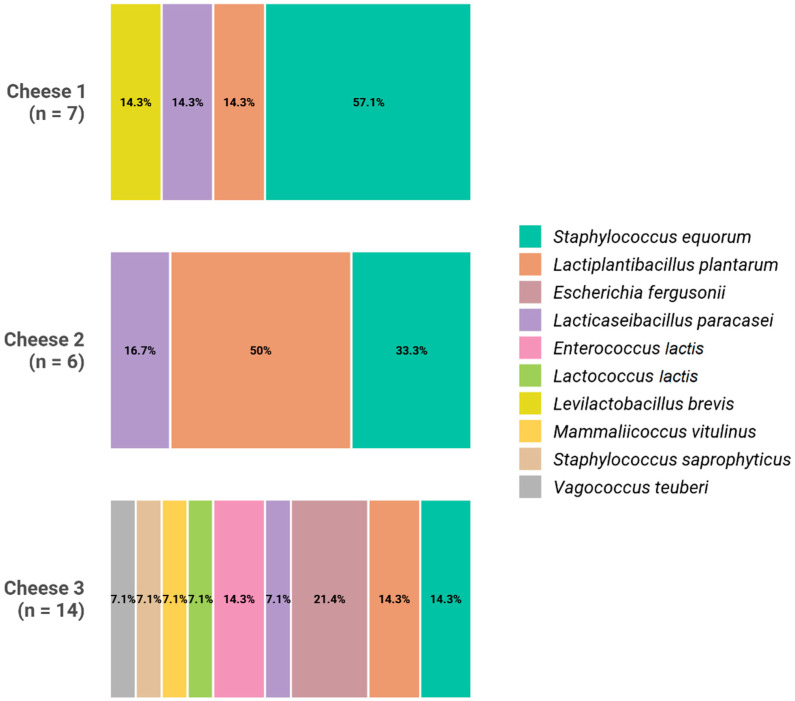
Distribution of bacterial identifications among the distinct colony morphologies observed in each cheese sample (Cheeses C1–C3). Stacked bars show the percentage of the total morphotypes analyzed (n: number of distinct morphologies characterized per cheese). The figure shows the results from one of the cheeses of each type although the same number of different isolates were obtained from all the samples within the same cheese type.

Clear differences were observed in the composition of the cultivable microbiota when the three types of cheeses were compared (Figure 3). A total of seven morphologically different isolates were isolated from the C1 cheeses, produced using the commercial starter alone. Among them, four isolates were identified as *Staphylococcus equorum* while each of the remaining three isolates belonged to *Lactiplantibacillus plantarum*, *Lacticaseibacillus paracasei* and *Levilactobacillus brevis*. A total of six different isolates were retrieved from the C2 cheeses, elaborated with the commercial starter and *L. salivarius* SP36. Subsequently, they were identified as *S. equorum* (two isolates), *L. plantarum* (three isolates) and *L. paracasei* (one isolate). Finally, the C3 cheeses, produced using only *L. salivarius* SP36, exhibited the highest diversity of species. Among the 14 different isolates obtained from these cheeses, nine bacterial species were identified including *Escherichia fergusonii* (three isolates), *S. equorum* (two isolates), *L. plantarum* (two isolates), *L. paracasei* (one isolate), *Lactococcus lactis* (one isolate), *Enterococcus lactis* (two isolates), *Mammaliicoccus vitulinum* (one isolate), *Staphylococcus saprophyticus* (one isolate), and *Vagococcus teuberi* (one isolate). *L. plantarum* and *L. paracasei* were the predominant species in the three types of cheeses, reaching mean values of approximately 4.0, 5.4 and 6.0 log_10_ CFU/g in the C3, C1 and C2 cheeses respectively.

Although *L. salivarius* isolates were not obtained from any sample using culture-dependent methods, quantitative PCR allowed the detection of *L. salivarius* DNA from the samples of the C2 and C3 cheeses, with mean values of 3.29 and 2.97 log_10_ copies/g, respectively.

The antibiotic susceptibility of the different LAB isolates was determined using E-test strips, and the minimum inhibitory concentration (MIC) values obtained for each antibiotic are shown in Table 3. Overall, the MICs for most of the *L. paracasei* and *L. plantarum* isolates were within the threshold currently required by EFSA [28], with some exceptions. Among the six *L. plantarum* isolates, four of them displayed MIC values against ampicillin (3–4 mg/L) slightly higher than the EFSA threshold (2 mg/L). Two of the three *L. paracasei* isolates were resistant to chloramphenicol although, again, its MIC values (6 mg/L) were close to their corresponding EFSA threshold (4 mg/L). The same two *L. paracasei* isolates showed resistance to kanamycin (MIC 128 mg/L; threshold: 64 mg/L). Although EFSA [28] does not require testing sensitivity against vancomycin for *L. paracasei*, *L. plantarum* and *L. brevis* isolates because of intrinsic resistance, the E-test assay confirmed that all the isolates belonging to these species were resistant to this antibiotic (MIC values > 24 mg/L). In the case of the lactococcal and enterococcal isolates, all the MIC values were within the EFSA threshold.

None of the LAB *Enterococcus lactis* and *Escherichia fergusonii* isolates showed an ability to form biogenic amines, including tyramine, histamine, putrescine, and cadaverine.

### 3.4. Preliminary Analysis of the Genomes of L. plantarum Q132 and L. paracasei Q133

A genome-based safety screening of the two strains isolated from cheese, *L. plantarum* Q132 and *L. paracasei* Q133, based on the combined Bakta and RAST annotation sets, did not identify genes encoding classical foodborne toxins, and no canonical gene clusters classically associated with the production of biogenic amines were detected in the annotated dataset. A CARD analysis identified one strict hit in Q132 corresponding to a *vanY*-like determinant associated with the vancomycin resistance gene cluster, classified under glycopeptide antibiotic resistance by target alteration, whereas in Q133 a single strict hit corresponding to *qacJ* was detected, annotated as a small multidrug resistance (SMR) efflux pump associated with disinfecting agents and antiseptics, including benzalkonium chloride.

### 3.5. Metataxonomic Analysis of the Cheeses

**Figure 4:**
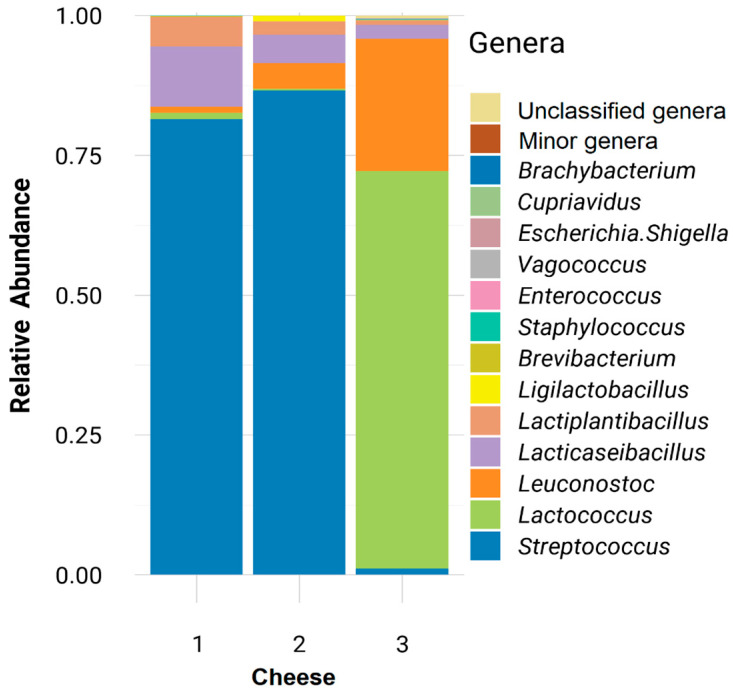
Relative abundance profiles, at the genus level, of the three types of cheeses elaborated in this study. The data represent the mean of the three cheeses of each cheese. The stacked bars represent the proportional composition of bacterial genera in each cheese type (1, C1; 2, C2; 3, C3). Low-abundance taxa are grouped as “Minor genera” while reads not confidently assigned are grouped as “Unclassified genera”.

Three samples of each type of cheese were submitted to a 16S RNA-based metataxonomic analysis. The relative abundance of the genus *Streptococcus* was very high in the two cheeses elaborated with the commercial starter. The means (±SD) were 81.5 ± 1.6 and 86.6 ± 0.45 in the C1 and C2 cheeses, respectively (Figure 4; Table S1), while in the C3 cheese (without the commercial starter) was 1.2 ± 0.04. In the C3 cheese, *Lactococcus* was the most abundant genus (71.0 ± 0.2), followed by the genus *Leuconostoc* (23.7 ± 0.09). The genera *Lacticaseibacillus* and *Lactiplantibacillus* were detected at relatively high abundances in the two cheeses in which the commercial starter was used (10.8 ± 1.6 and 5.2 ± 0.2, respectively, in the C1 cheese, and 5.1 ± 0.3 and 2.4 ± 0.1, respectively, in the C2 cheese). Both genera were also detected, although at a lower abundance, in the C3 cheese (2.5 ± 0.1 and 0.8 ± 0.07, respectively) (Figure 4; Table S1). *Ligilactobacillus* sequences were only detected in the C2 and C3 cheeses, in which *L. salivarius* SP36 was added, although its relative abundance was low (1.0 ± 0.05 and 0.04 ± 0.007, respectively). Sequences belonging to the genera *Staphylococcus*, *Brevibacterium* and *Cupriavidus* were also retrieved from the three cheeses at low or very low relative abundances. *Enterococcus* DNA was present in the C1 and C3 cheeses while sequences belonging to the genera *Escherichia-Shigella*, *Vagococcus* and *Brachybacterium* were only detected in C3 samples at very low relative abundances (Figure 4; Table S1).

## 4. Discussion

In this study, the effect of *L. salivarius* SP36, either alone or in combination with a commercial starter, on some microbiological and physico-chemical properties of cheese after a 150-day maturation period was assessed. Previously, it was found that this strain had promising properties as an adjunct culture in Manchego cheese [22]. The strain was isolated from a cheese seal that was last used in 1936 in a small Spanish Pyrenees village, which was abandoned in the middle 60s [21]. This work has the peculiarity of evaluating this strain in a small artisanal dairy whose location (Morillo de Sampietro, Huesca, Spain) shares the same geographical and ecological conditions as the dairy where the seal was routinely used for decades.

The culture-based analysis revealed the absence of *Salmonella* spp., *L. monocytogenes* and coagulase-positive staphylococci in the three types of cheeses, characterizing them as microbiologically safe according to EU legislation. However, some *S. equorum*, *M. vitulinus*, *S. saprophyticus*, *E. lactis*, *V. teuberi* and *E. fergusonii* isolates were isolated from the selective media employed to detect the potential pathogens cited above. It has been reported previously that Gram-positive catalase-positive cocci, including *S. equorum*, *S. saprophyticus* and *M. vitulinus* (known as *Staphylococcus vitulinus* until 2020 [34]) are frequently isolated from sheep-derived fermented products [35], including cheeses and cheese brines [36,37,38,39]. *E. lactis* (previously included within the *Enterococcus faecium* species) is often isolated from raw milk cheeses [40] and, in fact, some *E. faecium* strains are used as commercial adjunct cultures [41]. Finally, the presence of *V. teuberi* [42] and *E. fergusonii* [43] in artisanal cheeses or fermented milks have also been described, although at a much lower frequency. The C3 cheeses had the highest culturable microbial diversity, probably because of the lack of the commercial starter culture responsible for rapid acidification and the inhibition of acid-sensitive bacteria. Our results suggest that the metabolic activity of the starter bacteria may enhance *Ligilactobacillus* performance since both the quantitative PCR and the metataxonomic approaches revealed that its abundance in the C3 cheeses was lower than in the C2 cheeses.

Among LAB, isolates of *L. plantarum* and *L. paracasei* were obtained from the three types of cheeses, while *Lactiplantibacillus* and *Lacticaseibacillus* DNA were also relatively abundant in all the cheeses. These two species are common and often dominant members of non-starter LAB communities in cheeses [44,45,46]. Both may be present at low concentrations (even below the detection level) in the curd but their concentrations may increase sharply during ripening [22,46] and have big potential to be used as adjunct or biopreservative cultures [47,48,49].

In relation to antibiotic resistance, *L. plantarum* Q132 and *L. paracasei* Q133 fulfilled the current criteria of EFSA [28] for microorganisms intended to use in the food chain. All the *L. plantarum* and *L. paracasei* isolates obtained from the three types of cheeses were resistant to vancomycin; however, this is not a limitation for their use in cheesemaking since the composition of the peptidoglycan precursors of these species confers intrinsic resistance to this antibiotic [50]. Four *L. plantarum* isolates displayed resistance against ampicillin while two *L. paracasei* isolates were resistant to chloramphenicol and kanamycin. Although the MIC values were very close to their respective thresholds and intrinsic resistance toward these antibiotics have been found among LAB isolated from foods [51], only *L. plantarum* Q132 and *L. paracasei* Q133 were selected for future applications and submitted to an additional preliminary characterization, including WGS and the potential production of biogenic amines. Mining the *L. plantarum* Q132 and *L. paracasei* Q133 genomes revealed that they do not harbor transmissible antibiotic resistance genes.

Biogenic amines may be present in fermented foods, such as cheese, because of the activity of bacteria that are able to decarboxylate precursor amino acids [52]. However, these compounds are associated with adverse effects on human health [53]. Consequently, an inability for biogenic amine production should be included among the criteria for selecting strains used as either starter or adjunct cultures in cheesemaking. The phenotypic and genomic results of this study indicate that *L. plantarum* Q132 and *L. paracasei* Q133 are unable to form biogenic amines.

The metataxonomic approach showed a low relative abundance of *Ligilactobacillus* sequences in the C2 and C3 cheeses after 150 days of ripening, despite *L. salivarius* SP36 being initially inoculated at a high dose. Quantitative PCR analyses detected *L. salivarius* sequences in these two types of cheeses although, again, at low densities (3.29 and 2.97 log_10_ CFU/g in the C2 and C3 cheeses, respectively). Such a low abundance may explain why none of the isolates obtained on MRS agar plates belonged to this species since the concentration of other LAB species (mainly *L. paracasei* and *L. plantarum*) were approximately 2–3 log_10_ units higher. Despite the low abundance of *L. salivarius* in the C2 and C3 cheeses at the end of the ripening period, the use of *L. salivarius* SP36 as an adjunct culture may exert a noticeable effect on the cheeses. In a previous work, the administration of this strain as an adjunct culture to Manchego cheeses led to significant changes in the profile of volatile compounds and, also, in the organoleptic properties in comparison to control cheeses elaborated using a commercial starter [22]. However, its relative abundance after 120 and 240 days of ripening was low, ranging between 1.12 and 2.24%. In other work, the concentration of *L. salivarius* MP101 in a fermented milk decreased sharply after 42 days at 2–8 °C [54]. The poor fermentative competitiveness of *L. salivarius* strains in dairy substrates may be due to the presence of a GalR-LacI repressor and the absence of genes encoding caseinolytic proteinases in its genome [21]. This fact may be responsible for the low survival and colonization observed in this work.

However, *L. salivarius* SP36 may contribute to the biosynthesis of volatile compounds during ripening through two mechanisms. The first is its own proteolytic and lipolytic activities, as previously reported when the strain was added as an adjunct culture to Manchego cheeses [22]. The WGS of this strain revealed that its genome harbors a wide array of genes involved in the catabolism of other proteins, peptides, and amino acids, as well as enzymes responsible for flavor development, including aromatic amino acid aminotransferase gamma and quinate 5-dehydrogenase I beta [21]. Its genome also contains several genes related to lipolysis, including phosphoesterase, cyclic-di-AMP phosphodiesterase, glycerophosphoryl diester phosphodiesterase, and thioesterase [21]. The second is its ability to promote the growth of autochthonous non-starter lactic acid bacteria (*L. plantarum* and *L. paracasei*). In a different context, it has been previously shown that the administration of two different *L. salivarius* to women, at a daily dose of 1–3 × 10^9^ ufc for up to 6 months, led to their presence at relatively low concentrations in the respective vaginal samples; interestingly, the administration of such strains allowed a big increase in the density of autochthonous vaginal lactobacilli, such as *L. crispatus* or *L. jensenii* [27,55,56].

The metataxonomic analysis revealed the presence of DNA belonging to genera that were also detected by culture-based methods (*Lactiplantibacillus*, *Lacticaseibacillus*, *Staphylococcus*, *Enterococcus*, *Lactococcus*, *Vagococcus* or *Escherichia*). However, a major taxonomic inconsistency was also detected between culture-based and metataxonomic analyses since the last approach showed that genus *Streptococcus* dominated the C1 and C2 cheeses despite being elaborated by using a *Lactococcus*-based commercial starter. Such inconsistencies may be related to methodological issues, a fact that highlights the relevance of culture-based approaches when assessing the microbiological composition of cheeses.

In contrast, the relative abundance of the genus *Lactococcus* was very low in the C1 and C2 cheeses. When inoculated as a starter culture, *Lactococcus lactis* is the most abundant species in the cheese at the beginning of the maturation period; however, its concentration decreases as the ripening period increases [22]. Interestingly, a lactococcal isolate was recovered from the C3 cheese samples while, at the genus level, *Lactococcus* DNA was the most abundant in the same cheese. Wild *L. lactis* strains have been previously isolated from cheeses with excellent sensorial properties [57,58].

The pH of the C3 cheeses was significantly higher (~6.3) than that of the C1 and C2 cheeses (~5.6). A previous study [21] showed that *L. salivarius* SP36 was able to produce high amounts of L-lactic acid (~11.1 mg/mL) when growing in MRS broth, leading to a rapid decrease in pH (~3.94). In contrast, the concentration of L-lactate was much lower when this strain grew in milk (~0.21, pH = 5.97). These results suggest that, while the strain may be used as an adjunct culture for cheesemaking, its low milk acidification capacity may not be sufficient to serve as a single starter culture. Although the *L. salivarius* SP36 genome harbors L-lactate dehydrogenase genes (*ldhL*) and contains the genes encoding all the enzymes required to metabolize lactose predominantly through the Leloir pathway, it also contains a galactose operon repressor. Interestingly, *L. salivarius* is a species that has been isolated from the fresh milk of different mammalian species, and it has been shown that it can colonize the mammary gland during late pregnancy and throughout the lactation period [59]. In the intra-mammary environment, the acidification and subsequent coagulation of milk would not be a desirable trait and this may be the reason for the presence of such a repressive mechanism.

Additional differences were found among the cheeses in relation to water activity, which was lower in the C3 cheeses than in the C1 and C2 cheeses, and to lightness, which was lower in the C2 cheeses when compared to the other two types of cheeses. A variety of factors exert an influence on water activity (microbial metabolism, cheese structure and composition, and ripening conditions) and color parameters (microbial pigments, proteolysis, and fat oxidation) but there is not a clear explanation for the differences observed in this work. Future work is needed to confirm if such changes are repetitive and, in that case, to address the factors responsible for them.

In relation to the impact of adding *L. salivarius* SP36 on the volatile profiles of the cheeses, in a previous study, cheeses made with this strain but with a different commercial starter also showed higher levels of esters than the control cheese made only with the starter culture [22]. These differences in ester concentration were attributed to variations in LAB composition. It has been reported that *L. salivarius* strains isolated from human milk display esterase activities on most tested substrates when evaluated for dairy technological properties [60]. LAB esterases catalyze the synthesis of esters from glycerides and alcohols via alcoholysis during cheese ripening [61]. Esters, characterized by very low perception thresholds, play an important role in the aroma profile of different cheese varieties. Most esters commonly found in cheese are associated with sweet, fruity and floral notes [62,63,64,65,66].

Consistent with our results, Arias et al. [22] also reported higher levels of alcohols in cheeses manufactured with *L. salivarius* SP36 than in control cheeses. In general, primary alcohols have small contributions to cheese aroma, mainly due to their high perception threshold, but they act as limiting factors in ester formation in hard cheeses [66]. Branched-chain primary alcohols, such as 2-methyl-1-propanol and 3-methyl-1-butanol, are formed by the reduction in the corresponding aldehydes derived from aspartic acid and leucine, respectively. These alcohols have fruity, fusel oil, or whisky-like odors, and even a chocolate flavor, depending on their concentration. Phenyl ethanol, a primary aromatic alcohol, originates from phenylalanine, and is among the most odorous aromatic alcohols, with a pleasant aroma associated with rose flower notes [62]. Secondary alcohols are formed by enzymatic reduction in the corresponding methyl ketones, which themselves are derived from fatty acids by β-oxidation or from β-ketoacids [62]. Fruity, fresh, herbaceous and ripened cheese odor notes have been described for these alcohols. 2-Heptanol has been identified as a key odorant of Gorgonzola and Grana Padano cheeses [62]. On the other hand, 2,3-butanediol can be produced by a redox reaction from acetoin and has been considered as a potential contributor to the sensory quality of Serpa cheese [67].

Methyl ketones are formed from fatty acids through a metabolic pathway connected to β-oxidation and play an important role in cheese aroma due to their much lower perception thresholds [46]. Acetoin (3-hydroxy-2-butanone) originates from diacetyl (2,3-butanedione) by reduction and has been described as imparting sour milk odor notes [62]. Adding *L. salivarius* AR809 to Monascus-ripened cheese increased methyl ketone production and reduced certain ethyl esters, enhancing flowery, fruity, and musty flavors [68].

The use of *L. salivarius* SP36 as an adjunct in cheese manufacture also promoted (*p* < 0.01) the formation of acetic and butanoic acids (Table 2). Similarly, Arias et al. [22] reported higher levels of acids in Manchego cheeses made with *L. salivarius* SP36 as an adjunct culture than in control cheese. Short-chain carboxylic acids, which have low perception thresholds, are major odorants in different cheese varieties. The use of *L. salivarius* AR809 as an adjunct in the manufacture of a mold-ripened cheese promoted the formation of alcohols and acids [20]. Higher levels of acetic acid were also found in cheeses made with two *L. salivarius* strains compared to their control cheese [68]. Acetic acid has a typical vinegar odor, while butanoic acid imparts a rancid cheese-like odor and plays an important role in the flavor of cheese varieties such as Camembert, Cheddar, Grana Padano, Gruyere, Pecorino, Ragusano and Roncal [62].

The C2 cheese, made with the starter culture and the strain SP36, showed higher (*p* < 0.01) relative abundances of hexane, octane, 2- and 3-octene, and 3,7-dimetyloctene compared to the other two cheeses (Table 2). Higher levels of some hydrocarbons have also been reported in cheeses made with *Lactobacillus helveticus* as an adjunct [69]. Hydrocarbons originate from lipid oxidation and are frequently detected in the volatile fraction of cheeses, usually at low concentrations not detectable by olfactometry.

Levels of dimethyl sulfide were higher (*p* < 0.01) in the C2 cheese, while dimethyl sulfone levels were higher in the two cheeses including *L. salivarius* SP36 (Table 2). Sulphur compounds essentially originate from methionine degradation and play an important role in cheese flavor due to their very low perception thresholds [62]. These compounds are generally associated with sulfurous, cabbage-like, hot milk, and burnt odor notes.

Finally, the C2 cheese showed higher levels of α-pinene than the other two cheeses (*p* < 0.01; Table 2). This compound is among the most frequently identified terpenes in the volatile fraction of cheeses. Terpenes originate from the plants that constitute the forage mixture of the pastures [62]. α-Pinene has been associated with pine and green odor notes.

This study has the advantage of using the *L. salivarius* SP36 in an artisanal dairy with the same environmental conditions of ancient cheese-making in the Spanish Pyrenees. Conversely, it also faces the limitation of the small quantity of cheeses that can be made and matured in this small dairy. Therefore, the number of cheeses that were evaluated in this study was very low and it may affect the reproducibility and representativeness of our results. However, this study confirms the ability of *L. salivarius* SP36 to modify the biochemical properties of the matured cheeses, which was already proven in a different ecological context [22]. This paves the way for the commercial use of this strain as an adjunct culture, which is already in progress in the same dairy that participated in this work. In addition, work is also in progress to elucidate the potential roles as adjunct or biopreservative cultures of the *L. plantarum*, *L. paracasei* and *L. lactis* isolates obtained in this study.

## 5. Conclusions

The use of *L. salivarius* SP36 as an adjunct in cheese manufacture promoted the formation of numerous key volatile flavor compounds, including esters, alcohols, ketones, and acids. In contrast, *L. salivarius* SP36 alone had a comparatively minor effect on the formation of these compounds, suggesting metabolic complementarity and cooperation between *L. salivarius* SP36 and starter LAB in the formation of the volatile compounds. Future studies should address the impact of using this strain in small artisanal dairies on the organoleptic properties of the cheeses. The biotechnological potential of the *L. plantarum*, *L. paracasei* and *L. lactis* isolates obtained in this study should be addressed in subsequent studies.

## Figures and Tables

**Table 1:** Physico-chemical parameters of the cheeses elaborated in this study. Values are expressed as mean ± standard deviation.

Parameter	C1 (Starter)	C2 (Starter + SP36)	C3 (SP36)	*p*-Value
pH	5.69 ± 0.05 a	5.58 ± 0.09 a	6.35 ± 0.10 b	0.0001
a_w_	0.91 ± 0.006 a	0.90 ± 0.004 a	0.88 ± 0.003 b	0.0006
L*	75.65 ± 1.56 a	67.89 ± 4.18 b	75.09 ± 2.29 a	0.0002
a*	−4.38 ± 0.25 a	−5.09 ± 0.48 b	−4.39 ± 0.19 a	0.0050
b*	21.48 ± 0.66 a	21.09 ± 1.20 a	21.87 ± 0.63 a	0.1489

*p*-values were calculated using one-way ANOVA; means in the same row followed by different letters differ significantly (*p* < 0.05).

**Table 2:** Volatile compounds significantly affected using *Ligilactobacillus salivarius* SP36 in cheeses.

	C1 (Starter)	C2 (Starter + SP36)	C3 (SP36)
Hydrocarbons			
Hexane	0.64 ± 0.13 a	1.27 ± 0.20 b	0.47 ± 0.07 a
Octane	0.34 ± 0.07 a	2.11 ± 0.13 b	0.50 ± 0.19 a
3-Octene	0.53 ± 0.07 a	5.14 ± 0.51 b	1.10 ± 0.16 a
2-Octene	0.50 ± 0.18 a	2.15 ± 0.13 b	0.75 ± 0.30 a
3,7-Dimethyloctene	2.89 ± 0.73 a	4.50 ± 0.04 b	2.58 ± 0.32 a
Sulphur compounds			
Dimethyl sulfide	0.04 ± 0.02 a	0.32 ± 0.05 b	0.11 ± 0.07 a
Dimethylsulfone	4.35 ± 0.59 a	6.52 ± 0.48 b	6.99 ± 0.87 b
Benzene compounds			
Alpha-pinene	8.81 ± 1.08 a	17.48 ± 2.41 b	12.01 ± 0.94 a
Phenyl ethanol	3.09 ± 0.57 c	2.16 ± 0.18 b	1.30 ± 0.17 a
Ketones			
2-Pentanone	84.16 ± 4.24 a	80.23 ± 6.67 a	143.19 ± 30.94 b
Acetoin	94.65 ± 8.76 a	74.76 ± 6.26 a	246.61 ± 36.12 b
Esters			
Ethyl acetate	14.44 ± 1.48 a	34.26 ± 1.71 b	21.74 ± 5.50 ab
Ethyl butanoate	92.22 ± 14.74 a	232.24 ± 20.61 b	156.38 ± 42.29 ab
Ethyl-2-methylbutyrate	9.54 ± 2.61 b	4.61 ± 1.30 a	16.12 ± 2.18 c
Ethyl hexanoate	33.37 ± 4.17 a	82.15 ± 5.98 b	75.68 ± 28.75 ab
Ethyl lactate	1.56 ± 0.28 a	4.63 ± 0.69 b	2.33 ± 0.48 a
Ethyl octanoate	2.02 ± 0.25 a	6.09 ± 0.89 b	3.65 ± 1.23 a
Alcohols			
2-Propanol	2.68 ± 0.54 a	5.83 ± 0.57 b	2.30 ± 0.62 a
Ethanol	53.05 ± 14.10 a	165.77 ± 10.13 b	74.78 ± 24.19 a
2-Methyl-1-propanol	5.33 ± 0.94 a	7.19 ± 1.28 ab	11.54 ± 2.85 b
1-Methoxy-2-propanol	2.74 ± 0.50 a	2.98 ± 0.18 ab	4.07 ± 0.55 b
2-Pentanol	12.25 ± 0.88 a	41.71 ± 5.16 b	18.61 ± 5.50 a
1-Butanol	1.11 ± 0.03 a	2.95 ± 0.43 c	2.09 ± 0.18 b
3-Methyl-1-butanol	18.07 ± 0.63 a	23.68 ± 3.35 ab	33.79 ± 6.50 b
2-Heptanol	2.59 ± 0.94 a	6.35 ± 1.88 b	3.17 ± 11.14 a
1,3-Butanediol	46.06 ± 10.20 b	79.00 ± 9.16 c	14.68 ± 1.01 a
2,3-Butanediol	37.24 ± 0.58 a	66.27 ± 3.53 b	61.47 ± 8.21 b
Acids			
Acetic acid	84.85 ± 22.70 a	217.28 ± 32.84 b	100.67 ± 16.48 a
Butanoic acid	241.68 ± 47.02 a	342.06 ± 24.25 b	248.73 ± 24.76 a

Results represented as mean (n = 4) ± SD of duplicate determinations in two cheeses, expressed as relative abundances to internal standard cyclohexanone. *p*-values were calculated using one-way ANOVA. Means in the same row followed by different letters differ significantly (*p* < 0.01).

**Table 3:** MICs (mg/L) of the different antibiotics required by EFSA for the LAB isolates obtained in this study.

Isolate	ERY	TET	GEN	CLI	STR	KAN	AMP	CHL	VAN
*Lacticaseibacillus paracasei* Q133	0.064	1.5	4	0.047	32	32	2	4	nr
*Lacticaseibacillus paracasei* Q231	0.032	2	2	0.032	32	128	1.5	6	nr
*Lacticaseibacillus paracasei* Q333	0.032	1	4	0.016	24	128	1.5	6	nr
*Lactiplantibacillus plantarum* Q132	0.094	32	0.075	0.016	nr	8	2	8	nr
*Lactiplantibacillus plantarum* Q230	0.094	32	0.75	0.016	nr	12	4	8	nr
*Lactiplantibacillus plantarum* Q232	0.125	32	0.75	0.016	nr	12	3	8	nr
*Lactiplantibacillus plantarum* Q233	0.19	24	1	0.016	nr	16	1.5	8	nr
*Lactiplantibacillus plantarum* Q330	0.125	32	0.38	0.016	nr	8	3	8	nr
*Lactiplantibacillus plantarum* Q332	0.094	32	0.75	0.016	nr	6	4	6	nr
*Levilactobacillus brevis* Q130	0.016	8	0.05	0.125	16	24	1	4	nr
*Lactococcus lactis* Q331	0.032	1.5	2	1	24	4	0.19	2	1.5
*Enterococcus lactis* Q334	2	16	3	0.064	128	nr	1.5	8	1.5

Abbreviations: nr, not required by EFSA [28]; ERY, erythromycin; TET, tetracycline; GEN, gentamicin; CLI, clindamycin; STR, streptomycin; KAN, kanamycin; AMP, ampicillin; CHL, chloramphenicol; VAN, vancomycin.

## Data Availability

The data presented in this study are openly available in GenBank under the accession number PRJNA1442380.

## Supplementary Materials

The following supporting information can be downloaded at https://www.mdpi.com/article/10.3390/foods15081362/s1, Table S1: Genus-level relative abundance data of the cheese microbiota obtained by metataxonomic analysis.

## Abbreviations

The following abbreviations are used in this manuscript:

LAB	Lactic Acid Bacteria
EFSA	European Food Safety Authority
