# Supplementary material for: Use of Ligilactobacillus salivarius SP36 as an Adjunct Culture by an Artisan Dairy and Isolation of New Autochthonous Strains with Technological Potential for Cheesemaking
Source: Foods. 2026 Apr 14;15(8):1362. doi: 10.3390/foods15081362 (PMC13114453; doi:10.3390/foods15081362)
Supplement: Supplementary file 1 [file foods-15-01362-s001.zip › Foods_Cheese_TableS1.pptx]

## Slide 1
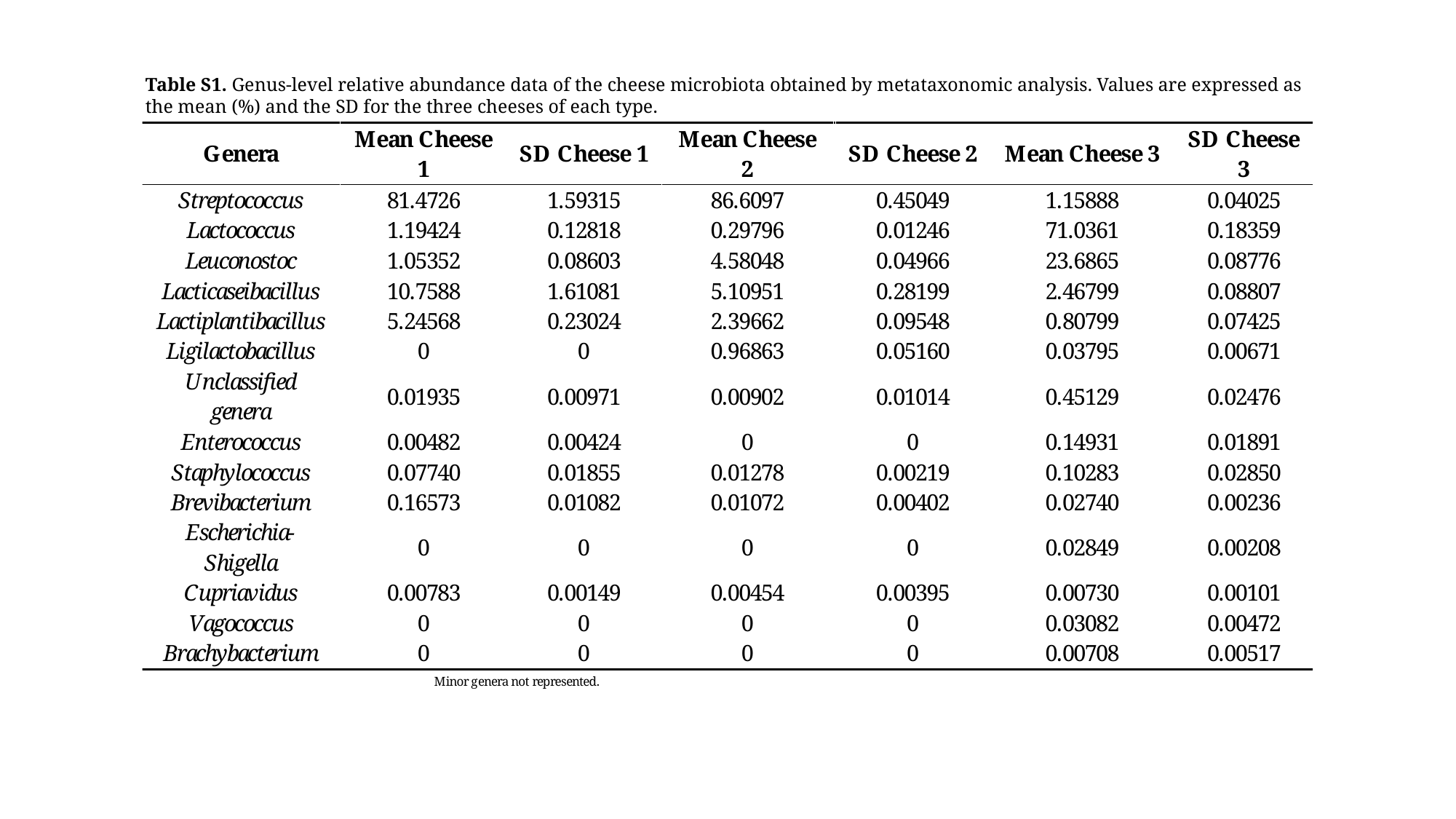

Table S1. Genus-level relative abundance data of the cheese microbiota obtained by metataxonomic analysis. Values are expressed as the mean (%) and the SD for the three cheeses of each type.
